# MYB transcription factors as context-dependent regulatory modules for precision crop improvement

**DOI:** 10.3389/fpls.2026.1836658

**Published:** 2026-04-20

**Authors:** Jing Zhang, Haibo Wu, Yubin Liu, Yunpeng Cao

**Affiliations:** 1 School of Health and Nursing, Wuchang University of Technology, Wuhan, China; 2 Guangxi Colleges and Universities Key Laboratory for Cultivation and Utilization of Subtropical Forest Plantation, Guangxi Key Laboratory of Forest Ecology and Conservation, School of Forestry, Guangxi University, Nanning, China

**Keywords:** context-dependent regulation, crop improvement, MYB transcription factors, pleiotropy, precision breeding

## Introduction

MYB transcription factors (TFs) are among the largest and most functionally diverse regulatory families in plants (Cao et al., 2020; Zhang et al., 2025a). Over the past two decades, they have been associated with a wide range of biological processes, including secondary metabolism, epidermal differentiation, cell-cycle regulation, reproductive development, and responses to abiotic and biotic stresses (Pratyusha and Sarada, 2022; Cao et al., 2023; Jin et al., 2024). Recent studies further emphasize that MYB proteins should not be viewed simply as regulators of isolated pathways, but rather as central components of broader regulatory networks that integrate development, metabolism, and environmental adaptation (Yu et al., 2023; Bhatt et al., 2025; Ma et al., 2025b; Wang et al., 2025; Zhang et al., 2025a). Historically, MYB research was strongly shaped by studies on visible phenotypes such as anthocyanin accumulation, flavonoid biosynthesis, and trichome formation (MaChado et al., 2009; Xu et al., 2015; Du et al., 2025), which were highly informative because they established MYBs as tractable models for transcriptional regulation and revealed the importance of combinatorial control in plants. However, the field has now expanded far beyond this initial framework. MYB factors are increasingly recognized as regulators of lignification, stress signaling, root development, cuticle formation, hormone responses, and developmental plasticity (Cao et al., 2016; Ma et al., 2025b; Zhang et al., 2025a). This expansion is particularly relevant under current agricultural challenges, where crop improvement must reconcile productivity, resilience, and quality under increasingly unstable environments.

At the structural and molecular levels, MYB proteins are defined by a highly conserved N-terminal DNA-binding domain (DBD), typically comprising one to four imperfect repeats (R1, R2, R3) that form helix-turn-helix structural motifs. Based on the genetic diversity and the number of these repeats, plant MYBs are phylogenetically classified into four major classes: 1R-MYB (MYB-related), R2R3-MYB, 3R-MYB, and 4R-MYB. Among these, the R2R3-MYB class is the most extensively expanded and functionally diverse in the plant kingdom (Caliskan et al., 2025; Zhang et al., 2025a). At the cellular level, MYBs regulate gene expression by recognizing specific cis-regulatory elements, such as MYB-binding sites (MBS), in the promoters of target genes. While the N-terminal DBD ensures DNA-binding specificity, the highly variable C-terminal region typically functions as a transcriptional activation or repression domain, often containing defined functional motifs such as the EAR (ERF-associated amphiphilic repression) motif. Furthermore, MYBs frequently exert their regulatory roles by forming multiprotein complexes, most notably the MYB-bHLH-WD40 (MBW) complex, which recruits chromatin modifiers and basal transcription machinery to orchestrate precise spatiotemporal gene expression (Xu et al., 2015).

It is important to acknowledge the functional heterogeneity within this massive gene family. While some MYBs act as highly specific “specialists” that regulate a single secondary metabolic pathway or a distinct developmental event, others function as pleiotropic integrators. In my view, the main significance of MYB TFs lies not only in the number of processes they regulate, but also in the type of biological control they represent. Plants constantly balance growth, defense, reproduction, and stress adaptation. Hub MYBs frequently act at these points of balance. For this reason, they are best understood as context-dependent regulatory modules that shape how plants allocate resources and prioritize biological functions. This Opinion argues that the MYB field has reached a stage at which descriptive studies alone are no longer sufficient. The next phase should focus on mechanistic interpretation, context dependence, and translational precision. Rather than continuing to ask only what *MYB* genes are present or whether one candidate affects one trait, the field should now ask how MYB-centered networks operate across tissues, developmental stages, and environmental conditions, and how these networks can be tuned for crop improvement without creating unacceptable trade-offs.

## Pleiotropic MYB transcription factors are regulatory hubs rather than simple pathway switches

MYB TFs are involved in far more than pigmentation or specialized metabolism. Zhang et al. (2025a) summarize roles of MYBs in phenylpropanoid metabolism, cell-cycle control, reproductive development, root hair formation, and multiple stress responses. Ma et al. (2025b) similarly highlight their importance in secondary metabolite biosynthesis and abiotic stress adaptation, while Bhatt et al. (2025) emphasize their broad involvement in plant development and defense-related pathways. This breadth changes how hub-type MYBs should be interpreted, as they are not merely peripheral regulators attached to individual traits, but rather frequently function at the intersection of developmental identity, metabolic allocation, and environmental responsiveness. MYB-mediated regulation of lignin, for example, influences cell wall properties, structural support, water transport, stress resistance, and biomass quality (Zhang et al., 2024, 2025b). Likewise, MYB-controlled flavonoid pathways affect pigmentation, antioxidant capacity, UV protection, and defense (Ye et al., 2024; Jiang et al., 2025). The same regulatory factor may therefore influence both physiological resilience and agronomic performance.

This integrative role is especially important in stress biology. MYBs associated with drought, salinity, cold, and heat responses often act through hormone-linked pathways, ROS homeostasis, osmotic adjustment, epidermal barriers, or root architecture (Ma et al., 2025b; Wang et al., 2025). These are not isolated outputs. They are components of coordinated survival strategies that often involve trade-offs with growth and reproduction. For that reason, pleiotropic MYB function should not be reduced to whether a gene is “positive” or “negative” for stress tolerance. A more informative question is how a specific MYB changes plant priorities under a given set of conditions.

## Strengths of current MYB research and where it remains limited

The current MYB field has several clear strengths. Comparative genomics has identified large MYB repertoires across many plant lineages and has clarified the evolutionary expansion of the family, especially the R2R3-MYB subgroup (Ma et al., 2025b; Zhang et al., 2025a). Functional studies in model species have revealed important mechanistic principles, including transcriptional complexes and pathway-specific regulation. In addition, transcriptomics, metabolomics, and transgenic analyses have linked MYB activity to measurable outputs in development and metabolism. However, despite significant advancements, current research on MYB transcription factors is constrained by several methodological and conceptual limitations. Primarily, the widespread reliance on descriptive workflows, typically progressing from phylogenetic classification to the isolated overexpression of candidate genes, often yields fragmented insights, failing to elucidate broader regulatory networks or tissue-specific mechanisms (Cao et al., 2023, 2025; Kong et al., 2026). Furthermore, the field exhibits a disproportionate emphasis on the R2R3-MYB subgroup, inadvertently neglecting other MYB classes that may govern essential developmental processes (Caliskan et al., 2025; Zhang et al., 2025a). Additionally, the prevalent use of constitutive overexpression for functional validation of pleiotropic MYBs can generate non-physiological artifacts that obscure critical growth trade-offs, making it an unreliable sole indicator of true agricultural breeding value. Finally, because MYB functions are intrinsically context-dependent, the heavy dependence on controlled laboratory and greenhouse studies limits our understanding of their dynamics in complex, multi-stress field environments, underscoring the need for more systemic and ecologically representative research approaches.

## MYB transcription factors are promising but difficult targets for crop improvement

From an applied perspective, MYBs are attractive targets because they regulate traits directly relevant to crop performance and quality. These include flavonoid and anthocyanin accumulation, lignin content, epidermal barrier formation, root development, and stress adaptation (Zhang et al., 2025a; Ma et al., 2025b; Bhatt et al., 2025). Because hub-type MYB transcription factors typically function upstream of entire genetic pathways, modifying a single MYB can simultaneously modulate coordinated sets of downstream genes. This characteristic offers a distinct advantage for the improvement of complex, polygenic traits in agriculture and biotechnology. Recent functional studies in major crops directly support this engineering paradigm while highlighting the associated trade-offs. For example, in soybean, the overexpression of *GmMYB14* improved both high-density yield and drought tolerance by modulating plant architecture and stress responses (Chen et al., 2021). In rice, *OsMYBS1* expression resulted in pleiotropic morphological changes that fortunately did not reduce total grain yield, showcasing successful multi-trait integration (Ma et al., 2025a). In maize, *ZmMYB92* modulates secondary wall cellulose synthesis, directly impacting stalk strength and biomass quality, which are crucial agronomic traits (Zhang et al., 2025b). Similarly, in woody crops like poplar, *PagMYB73A* enhances salt tolerance by facilitating adventitious root elongation, illustrating how MYBs coordinate developmental plasticity with environmental stress adaptation (Jin et al., 2024). Furthermore, in cotton, *GhMYB33* was identified as a critical hub gene mediating the growth-defense trade-off against the fungal pathogen *Verticillium dahliae* (Guang et al., 2024). However, altering these networks can also lead to conflicting outcomes. In horticultural crops like apple, different MYB family members exhibit antagonistic pleiotropic effects: *MdMYB305* promotes sugar accumulation but suppresses anthocyanin synthesis, whereas *MdMYB10* has the exact opposite effect, as empirically validated using both overexpression and CRISPR/Cas9 (Zhang et al., 2023). These crop-based examples underscore that the inherent hub-like nature of pleiotropic MYBs also presents a significant engineering challenge, as their perturbation frequently induces pleiotropic effects. For instance, engineering a MYB to enhance drought tolerance might inadvertently compromise overall plant biomass or delay developmental timelines. Similarly, manipulating MYBs to increase the accumulation of secondary metabolites, such as anthocyanin or lignin, can disrupt source-sink dynamics, biomass processing characteristics, and reproductive output. Ultimately, these phenotypic consequences are not merely unintended side effects; rather, they underscore the fundamental role that MYBs play in mediating essential biological trade-offs within the plant system.

The translational application of MYB transcription factors in crop improvement currently necessitates conceptual refinement to reach its full agricultural potential. Frequently, contemporary studies frame MYB genes as universal trait enhancers; however, pleiotropic MYBs are more accurately viewed as complex regulatory nodes that require precise tuning rather than binary activation or inactivation. In the context of drought resilience, for instance, MYBs function as molecular switches that integrate multiple signaling pathways, including abscisic acid (ABA) signaling, reactive oxygen species (ROS) scavenging, and broader metabolic processes (Wang et al., 2025). While this integration is vital for stress responses, it inherently risks pleiotropic effects, wherein enhanced drought tolerance may inadvertently compromise vegetative growth or yield under optimal conditions. Consequently, the ultimate metric for successful MYB manipulation is not merely the induction of a stress marker in short-term assays, but the enhancement of whole-plant performance across realistic, fluctuating environments. To bridge the gap between fundamental research and practical application, future studies must evaluate MYB-based strategies against rigorous agronomic endpoints, such as yield stability, biomass, flowering time, and fertility, ensuring these genetic modifications deliver viable, robust phenotypes in the field.

## The next phase should emphasize precision regulation and context-aware engineering

Due to their highly pleiotropic nature, hub MYB transcription factors are better suited for precision regulation rather than broad, constitutive manipulation. However, it is vital to note that this recommendation should be appropriately scoped based on the functional architecture of the target MYB. For trait-specific “specialist” MYBs—such as those exclusively controlling a single pigmentation step—traditional overexpression or knockout approaches remain perfectly valid and efficient for crop improvement. The choice of strategy must depend on whether the target MYB acts as an isolated switch or an integrated hub. Advancing our understanding of these hub regulators requires prioritizing spatiotemporal resolution, as their functions are deeply tissue-specific and stage-dependent, thereby necessitating the use of cell-type-informed and spatial transcriptomics (Luo et al., 2025). Furthermore, moving beyond mere expression correlation to identify direct regulatory mechanisms, such as specific cis-regulatory elements, protein partners, and chromatin dynamics, is critical for building predictive rather than merely descriptive regulatory networks. Additionally, because orthology does not guarantee equivalent functionality across species with diverse metabolic and structural architectures, rigorous crop-specific validation remains essential. Ultimately, future translational applications for pleiotropic MYBs must pivot from binary overexpression or complete knockouts toward nuanced regulatory tuning, utilizing targeted promoter editing, inducible expression, and tissue-specific control. By carefully modulating where, when, and to what extent MYB expression occurs, researchers can effectively harness their agricultural potential while minimizing undesirable biological trade-offs.

## Discussion

Recent literature leaves little doubt that MYB transcription factors are central regulators in plants, participating in and often connecting developmental patterning, secondary metabolism, cell wall formation, and stress responses rather than acting within a single domain alone (Zhang et al., 2025a; Ma et al., 2025b; Bhatt et al., 2025). Drought-focused analyses further support the view that MYBs function as integrators of multiple signaling pathways rather than isolated stress genes (Wang et al., 2025; Chen et al., 2026). The main challenge for the field is therefore no longer gene discovery, but interpretation and translation. MYB biology has already advanced beyond a simple catalog of family members. What is still needed is a predictive framework that explains how MYB-centered networks operate in specific developmental and environmental contexts, and how these networks can be manipulated with sufficient precision for crop improvement. As demonstrated by recent functional evidence in major crops, MYB transcription factors play central roles in plant growth, abiotic stress responses, and secondary metabolism. Future molecular breeding efforts should focus on precisely modulating MYB expression to improve stress tolerance, enhance nutritional quality, and ensure stable crop production under challenging environments. These points underscore the critical importance of mechanistic understanding and context-aware engineering—but without overgeneralizing across all MYBs. Our view is that MYB research should now become less descriptive and more mechanistic, less dependent on constitutive overexpression (particularly for hub MYBs), and more focused on context, trade-offs, and regulatory precision. Such a shift would not only improve the rigor of MYB studies, but also strengthen their practical relevance for agriculture. If this transition is achieved, MYB transcription factors may become one of the most useful regulatory platforms for designing crops that are more resilient, more efficient, and better balanced under variable environments.
